# What Do Participants of the Crohn's and Colitis UK (CCUK) Annual York Walk Think of Their Inflammatory Bowel Disease Care? A Short Report on a Survey

**DOI:** 10.1097/SGA.0000000000000261

**Published:** 2016-12-19

**Authors:** Antonina Mikocka-Walus, Madeleine Power, Lisa Rook, Gerry Robins, The CCUK York Walk Planning Committee

**Affiliations:** Antonina Mikocka-Walus, PhD, is Senior Lecturer, Department of Health Sciences, University of York, Heslington, York, United Kingdom.; Madeleine Power, MSc, is Research Assistant, Department of Health Sciences, University of York, Heslington, York, United Kingdom.; Lisa Rook, RN, is IBD Nurse, IBD Service, Department of Gastroenterology, York Teaching Hospital, York, United Kingdom.; Gerry Robins, MD, is Consultant Gastroenterologist, IBD Service, Department of Gastroenterology, York Teaching Hospital, York, United Kingdom.; CCUK York Walk Planning Committee, The Crohn's and Colitis UK York Walk Planning Committee, York, United Kingdom.

## Abstract

There has been a growing interest in a patient-centered model of care in inflammatory bowel disease; however, no relevant study using a mixed methodology has been conducted to date. Thus, our multidisciplinary group aimed to explore the issue of patient involvement in care among the inflammatory bowel disease community. A mixed-methods anonymous survey was conducted during the Crohn's and Colitis UK annual event. Summary statistics were used to describe the sample, and a simple thematic analysis identified key themes in qualitative responses. There were 64 survey respondents, representing 73% of the total family/friend groups participating (*N* = 87). Overall, 75% of respondents answered that they had the opportunity to discuss their care with their inflammatory bowel disease practitioner and 81% felt their opinions were taken on board and valued. A clear majority (84%) had at some point been treated by a gastroenterologist. In contrast, less than half (44%) had the opportunity for a dietician consultation and only 28% had the opportunity for a psychologist/counselor consultation. Although satisfaction with inflammatory bowel disease care was high, access to specialty services was concerning. Efforts should be made to provide access to mental health practitioners for those with clinically significant anxiety and/or depression.

## Introduction

There are 620,000 people living with inflammatory bowel disease (IBD) in the United Kingdom (UK). Up to 30% of IBD patients suffer comorbid anxiety and/or depression (Fuller-Thomson & Sulman, 2006; Walker et al., 2008), which, when left untreated, has been linked to more severe IBD symptoms and more frequent IBD flares (Mittermaier et al., 2004), higher hospitalization rates (van Langenberg, Lange, Hetzel, Holtmann, & Andrews, 2010), and lower compliance with treatment (Nigro, Angelini, Grosso, Caula, & Sategna-Guidetti, 2001) than for patients without these comorbidities. The current UK IBD Standards (TheIBDStandardsGroup, 2013) list the mental health of IBD patients as a particular challenge and an area for improvement.

## Background

Patient-centered care is gaining momentum in the National Health Service (National Institute for Health and Care Excellence [NICE], 2012), as it has a potential to improve both clinical and psychological outcomes. There has been a growing interest in a patient-centered model of care in IBD (Casellas-Jorda, Borruel-Sainz, Torrejon-Herrera, & Castells, 2012; Fontanet, Casellas, & Malagelada, 2004; Goodhand, Hedin, Croft, & Lindsay, 2011; Mawdsley, Irving, Makins, & Rampton, 2006; Mikocka-Walus, Turnbull, Holtmann, & Andrews, 2012; Pham, Grafton, van Langenberg, & Andrews, 2012; Sack et al., 2012; Torrejon Herrera et al., 2009; van Langenberg, Simon, Holtmann, & Andrews, 2010); however, no study exploring IBD patient views on patient-centered care using a mixed methodology has been conducted to date. Thus, our multidisciplinary group comprising a psychologist, health scientist, IBD nurse, gastroenterologist, and patients and caregivers explored the issue of patient involvement in care among the IBD community.

## Methods

A mixed-methods anonymous survey was conducted on May 17, 2015, during the Crohn's and Colitis UK (CCUK) Annual York Walk. The survey included simple demographics and questions on IBD care. Answering the survey implied consent, and this was made clear to participants. The questionnaire was distributed to the walkers on the event day as part of the registration and collected. One survey per group of walkers (typically comprising a family and friends of an IBD patient) was requested. A token of appreciation in the form of a bar of chocolate/candies was offered for returning a completed questionnaire. The collected data were entered into a database and analyzed. Summary statistics were used to describe the sample, and a simple thematic analysis (Braun & Clarke, 2006) identified key themes in qualitative responses. The study was approved by the University of York Health Sciences Research Governance Committee in March 2015.

## Results

### Respondent Characteristics

There were 64 survey respondents, representing 73% of the total family/friend groups participating (*N* = 87) and 20% of all the individual walkers (*N* = 325). The mean age was 37 years (*SD* = 17). Respondent demographic and clinical characteristics are presented in Table 1. Overall, 69% (*n* = 44) described themselves as an IBD patient, 9% (*n* = 6) as the parent of an IBD patient, 5% (*n* = 3) as the spouse/partner of an IBD patient, and 8% (*n* = 5) as “other carer for IBD patient.”

**TABLE 1.** Respondent Characteristics

Sample Characteristics	Respondents (*N* = 64)
Gender, female	42 (66%)
Race, White British	59 (92%)
Marital status, married/cohabitating	39 (61%)
Employment status, working full-time	31 (48%)
Education, tertiary	29 (45%)
IBD type, Crohn disease	40 (62%)
IBD duration, mean (SD), in years	9 (11)

*Note.* IBD = inflammatory bowel disease.

### IBD Care Providers

**FIGURE 1.**
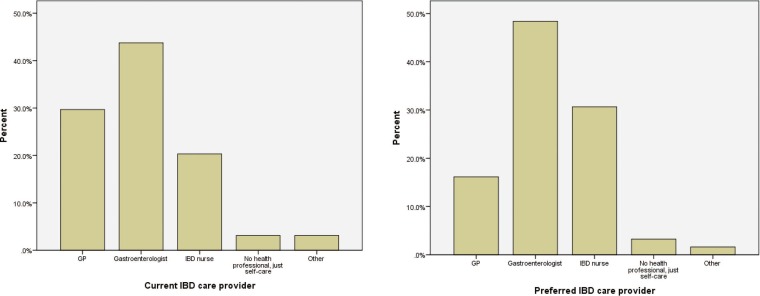
Current and preferred IBD care provider. IBD = inflammatory bowel disease.

Among the sample, the most common IBD healthcare provider was a gastroenterologist (*n* = 28; 44%) (Figure 1). The most common immediate point of contact for IBD flares, however, was an IBD nurse (*n* = 24; 37%) or a general practitioner (GP) (*n* = 19; 30%).

A clear majority (*n* = 54; 84%) had at some point been treated by a gastroenterologist. In contrast, less than half (*n* = 28; 44%) had the opportunity for a dietician consultation. Although only 28% (*n* = 17) had the opportunity for a psychologist/counselor consultation, 50% (*n* = 32) of respondents answered that although they had no opportunity for such a consultation, they did not think they needed one.

### Satisfaction With IBD Care

Responses suggest a high level of satisfaction, with a mean score of 7.7 (*SD* = 2) (a scale ranging from 0 to 10). Reflecting these high levels of satisfaction with their care, 75% (*n* = 48) of respondents answered that they had the opportunity to discuss their care with their IBD practitioner and 81% (*n* = 52) felt they had the ability to express their opinions to their IBD practitioner, and their opinions were taken on board and valued. Among respondents, the preferred care provider was a gastroenterologist (47%), whereas an IBD nurse was the second most preferred care provider (30%) (Figure 1). Only 10 respondents (16%) said that their GP was their preferred care provider.

### If You Had a Magic Wand, How Would You Change Your/the Person for Whom You Care's IBD Healthcare?

**FIGURE 2.**
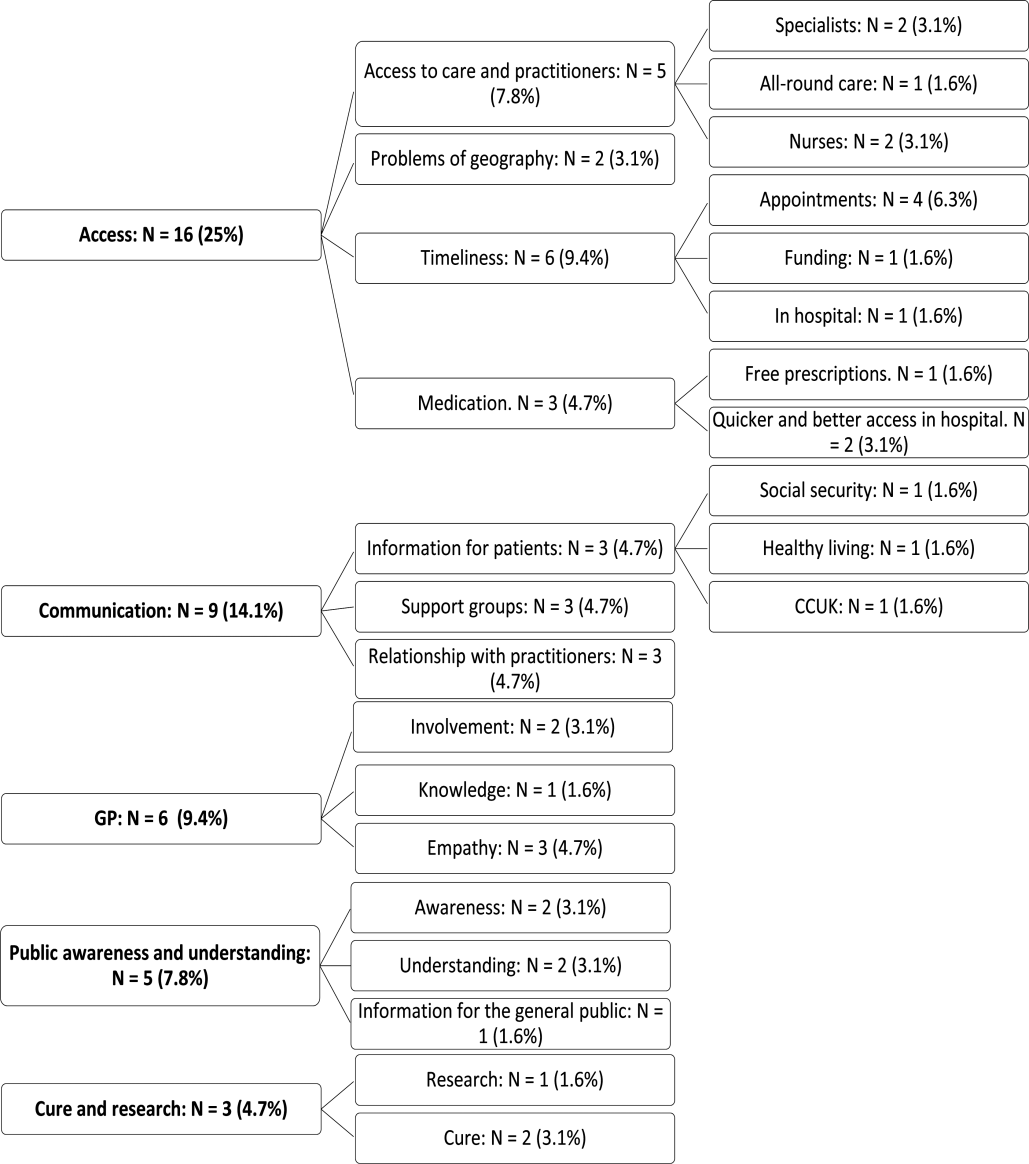
A thematic map of respondents' suggested improvements in inflammatory bowel disease care. CCUK = Crohn's and Colitis UK; GP = General practitioner.

Analysis of the qualitative data identified five common themes on how to improve IBD care: access; communication; GP services; public awareness and understanding; and cure and research (Figure 2).

#### Access

Better access to care was discussed by 16 (25%) respondents. Five respondents commented that they would like better access to practitioners in some form, including better and more frequent access to specialists such as gastroenterologists, dieticians, and psychologists, and more consistent and all-round care from practitioners. Access to care in terms of timeliness was cited by six respondents, with clear preference for shorter waiting times to see practitioners, when in hospital and for initial diagnosis, scans, and appointments. Two respondents highlighted geographical problems of access to their care provider, commenting on long journeys required to attend appointments. Three respondents called for better access to medication in some form, including “free prescription for all IBD medication in the UK” and when all available pain relief has been exhausted at home, for intravenous relief to be given on arrival at emergency services.

#### Communication

The need for improved communication between practitioners and patients, as well as better relationships more generally between healthcare services and patients and better information for patients, was discussed by nine respondents (14%). The need for good communication between care providers and patients was seen to be “key”; this required consideration, understanding, and listening on the part of practitioners. Better information about relevant social security benefits, lifestyle and healthy eating, local support groups, and the CCUK events was called for by four respondents, with one respondent citing the need for a 24-hour helpline.

#### GP Services

Six (9%) respondents commented on problems with the care provided by their GP. Comments addressed the low level of knowledge among GPs, the experience of lack of GP support and involvement with the patient's care, and the difficulties caused by their GP not being able to prescribe the patient's medication.

#### Public Awareness and Understanding

Five (8%) respondents discussed concerns about current level of public understanding and awareness about IBD. Respondents commented that there was a need for more information about IBD; one respondent suggested that better public awareness could quicken initial diagnosis.

#### Cure and Research

The need for a cure and the research required for this was touched upon by a small number (*n* = 3) of respondents.

## Discussion

This brief survey presenting patient views on IBD care in the UK (and Yorkshire specifically) contributes to the ongoing discussion on the optimal and most patient-friendly model of IBD care (Mikocka-Walus, Andrews, et al., 2012; Mikocka-Walus, Andrews, von Kanel, & Moser, 2013; Mikocka-Walus et al., 2014; Mikocka-Walus, Turnbull, et al., 2012).

The present study showed a high satisfaction with IBD care, with 75% of respondents reporting ability to discuss their care with an IBD practitioner and 81% reporting they had the ability to express their opinions to their IBD practitioner, with their views taken on board and valued. Despite this positive appraisal of the care received, access to care was still a significant concern (including better and more frequent access to psychologists and dieticians). Reflecting this, less than 30% of respondents had access to a psychologist. Although 50% of those who had access to a psychologist did not perceive a need to see one, there was also a substantial group of patients in need of psychological counseling who did not receive such care.

Given the high mental comorbidity rate in IBD (Fuller-Thomson & Sulman, 2006; Walker et al., 2008), efforts should be made to provide access to mental health practitioners for those with clinically significant anxiety and/or depression and to online psychotherapeutic resources, such as *Tame Your Gut* CBT program (http://www.tameyourgut.com/), for those with subclinical problems. Similarly, access to dieticians should be improved as the present survey showed that less than 50% of respondents had such access.

In line with the recent healthcare practitioners' survey on IBD care (Mikocka-Walus et al., 2014), the present survey's respondents preferred a gastroenterologist as their main IBD care provider, followed by an IBD nurse. Previous research demonstrated that GPs feel ill prepared to manage IBD (Tan, Holloway, Lange, & Andrews, 2012) and hence the present study provides yet another support for managing IBD in specialist-led gastroenterology clinics rather than in GP offices.

## Strengths and Limitations

The strength of this study lies in its mixed-methods approach, which allowed respondents the opportunity to share their views openly rather than rely on available survey options. Limitations of this study include its small sample size; however, the sample was a good representation of the family/friend groups who participated in the CCUK annual walk. The views of these respondents may nevertheless not be representative of all IBD patients and carers in the UK, and the findings can therefore be generalized only to those people who participate in events such as the CCUK annual walk. These could be the people who have their IBD sufficiently under control so that they are able to walk and people who are interested in raising IBD awareness. In fact, 62% of our respondents said their reason for participating in the event was to raise awareness of IBD or CCUK. Despite these limitations, this is one of very few studies exploring patient views on healthcare in IBD.

## Summary

This article demonstrates an unmet need for mental and dietary care as part of IBD management. Nurses are increasingly involved in IBD care around the world and considered to be the first point of contact in specialty clinics bearing responsibility for providing social support to patients (Mikocka-Walus et al., 2014). This puts nurses in a privileged position to be able to detect symptoms of mental disorders and either provide counseling themselves or refer patients to mental health providers. This article emphasizes an important role nurses may play in detection and treatment of mental symptoms associated with IBD.
